# N-of-1-*pathways* MixEnrich: advancing precision medicine via single-subject analysis in discovering dynamic changes of transcriptomes

**DOI:** 10.1186/s12920-017-0263-4

**Published:** 2017-05-24

**Authors:** Qike Li, A. Grant Schissler, Vincent Gardeux, Ikbel Achour, Colleen Kenost, Joanne Berghout, Haiquan Li, Hao Helen Zhang, Yves A. Lussier

**Affiliations:** 1 Center for Biomedical Informatics and Biostatistics, The University of Arizona, Tucson, AZ 85721 USA; 2 Bio5 Institute, The University of Arizona, Tucson, AZ 85721 USA; 3 Department of Medicine, The University of Arizona, Tucson, AZ 85721 USA; 4 Graduate Interdisciplinary Program in Statistics, The University of Arizona, Tucson, AZ 85721 USA; 5 Department of Mathematics, The University of Arizona, Tucson, AZ 85721 USA; 6 University of Arizona Cancer Center, The University of Arizona, Tucson, AZ 85721 USA; 7 Institute for Genomics and Systems Biology, The University of Chicago, Chicago, IL 60637 USA

**Keywords:** Precision Medicine, Single-Subject Analysis, N-of-1-pathways, Mixture Model, RNA-Seq, Head and neck squamous cell carcinomas (HNSCCs)

## Abstract

**Background:**

Transcriptome analytic tools are commonly used across patient cohorts to develop drugs and predict clinical outcomes. However, as precision medicine pursues more accurate and individualized treatment decisions, these methods are not designed to address single-patient transcriptome analyses. We previously developed and validated the N-of-1-pathways framework using two methods, Wilcoxon and Mahalanobis Distance (MD), for personal transcriptome analysis derived from a pair of samples of a single patient. Although, both methods uncover concordantly dysregulated pathways, they are not designed to detect dysregulated pathways with up- and down-regulated genes (bidirectional dysregulation) that are ubiquitous in biological systems.

**Results:**

We developed N-of-1-pathways MixEnrich, a mixture model followed by a gene set enrichment test, to uncover bidirectional and concordantly dysregulated pathways one patient at a time. We assess its accuracy in a comprehensive simulation study and in a RNA-Seq data analysis of head and neck squamous cell carcinomas (HNSCCs). In presence of bidirectionally dysregulated genes in the pathway or in presence of high background noise, MixEnrich substantially outperforms previous single-subject transcriptome analysis methods, both in the simulation study and the HNSCCs data analysis (ROC Curves; higher true positive rates; lower false positive rates). Bidirectional and concordant dysregulated pathways uncovered by MixEnrich in each patient largely overlapped with the quasi-gold standard compared to other single-subject and cohort-based transcriptome analyses.

**Conclusion:**

The greater performance of MixEnrich presents an advantage over previous methods to meet the promise of providing accurate personal transcriptome analysis to support precision medicine at point of care.

**Electronic supplementary material:**

The online version of this article (doi:10.1186/s12920-017-0263-4) contains supplementary material, which is available to authorized users.

## Background

Technologies, such as RNA-Seq, provide precise, timely, and cost-effective quantification of whole genome expression [1]. However, analytic tools remain underdeveloped for providing personal transcriptome profiling and individualized biological interpretation. Conventional transcriptome methods have been designed to uncover common mRNA and pathway signatures across a large cohort of patients, overlooking signals that differentiate one patient from another [2, 3]. The analysis of dynamic transcriptomes of a single subject has the potential to capture and inform gene expression changes reflective of personal physiological modifications, disease progression, and response to therapies in ways that genetic information cannot. Indeed, the majority of disorders with complex inheritance results from a combination of genetic risks and environmental factors unique to each patient that dynamically influence the course of disease. These dynamic biological changes that are genome X environment interactions between two conditions can be measured at the transcriptome level; however, current cohort-based statistics, which average signals across patients, are not applicable for the analysis of personal transcriptome dynamics [4]. Although in vitro assays were used to assess dynamic gene expression changes to predict experimental outcomes and disease progression at the patient level, these analyses remain limited and biased as they only assess a handful of gene candidates pertaining to known pathways [5]. However, scaling-up these assays and analyses to measure whole genome expression changes of a single subject (e.g., before and after treatment) has the advantage to unbiasedly discover dysregulated pathways unique to each individual.

Recognizing the limitations of conventional methods, we recently designed and validated in different disease contexts the N-of-1-*pathways*, which is a novel framework for single-subject transcriptome analysis based on a pair of samples (e.g., healthy and tumor, before and after therapy) from the same individual [6–10]. N-of-1-*pathways* relies on three principles: (1) the sole unit of observation is a single patient (case and control); (2) gene-level information are aggregated into gene sets (pathways); and (3) pathway results are summarized into personal biological profiling for clinical interpretation. Two methods under N-of-1-*pathways* framework were developed, N-of-1-*pathways* Wilcoxon (Wilcoxon) [6–8] using a Wilcoxon signed-rank test [11] and the N-of-1-*pathways* Mahalanobis distance (MD) [10, 12] using a statistical distance from a model of equal expression. The N-of-1-*pathways* Wilcoxon and MD analyze the dynamic change of mRNA expression and uncover dysregulated pathways (gene sets) from single-subject paired samples. The use of gene sets derived from gene ontology [13] provides computational advantage by reducing data dimension while providing mechanistic interpretation [14, 15]. While both methods have shown promise in single-subject transcriptome analysis, they were not designed to identify pathways (gene sets) with both up-regulated and down-regulated mRNA expressions and, therefore, take into account only concordantly dysregulated mRNAs within a pathway. In addition, Wilcoxon and MD are both self-contained methods [16] analyzing only mRNAs within a gene set and do not account for background noise due to technical and experimental artifacts [17–19].

To address the shortcomings of the current single-subject transcriptome analysis methods, we developed a novel approach within the N-of-1-*pathways* framework: N-of-1-*pathways* MixEnrich (MixEnrich) using a mixture model (mixture of two distributions: dysregulated vs. unaltered mRNAs) followed by a competitive-based [16] enrichment test. Self-contained (non-competitive) methods use exclusively the gene expression values of a gene set, while competitive methods utilize the entire transcriptome as a background [16]. MixEnrich is designed to cluster all mRNAs expression into two groups, unaltered and dysregulated (including up- and down-regulated), using mixture modeling [20]. Then pathways enriched with bidirectionally dysregulated mRNAs are identified using Fisher’s exact test [21]. Notably, this method builds on the work of Piccolo and his colleagues who have successfully applied mixture modeling in single samples for a different problem: to identify expressed vs. non-expressed mRNAs [22]. To test the performance of N-of-1-*pathways* MixEnrich in comparison to the only other single-subject paired-sample gene set tests (Wilcoxon and MD), we performed a simulation study and validation case study. We show that MixEnrich outperforms Wilcoxon and MD under various scenarios of simulated dysregulated pathways. This synthetic result was validated in a case study using head and neck squamous cell carcinomas (HNSCCs) RNA-Seq dataset, where MixEnrich uncovered biological relevant dysregulated pathways.

## Methods

### Datasets

#### Transcriptome datasets (Table 1)

**Table 1:** Dataset description

Dataset and Study	Dataset I: Simulation study	Dataset II: Validation case study 1	Dataset III: Validation case study 2
Type	Healthy lung tissues	Head and Neck squamous cell carcinomas	Breast invasive carcinoma
Source	TCGA	TCGA	TCGA
Date	March 2013	May 2015	October 2016
Platform	Illumina RNA-Seq V.2	Illumina RNA-Seq V.2	Illumina RNA-Seq V.2
Genes mapped	20,502	20,501	20,501
Patients			
Total	55	45 pairs	112 pairs
Healthy	55	45	112
Tumor	not applicable	45	112
URL	https://tcga-data.nci.nih.gov	https://tcga-data.nci.nih.gov	https://tcga-data.nci.nih.gov

An RNA-Seq dataset of 55 normal lung tissue samples from The Cancer Genome Atlas (TCGA) [23] was used to estimate expression means for each mRNA in the simulation study. To validate N-of-1-*pathways* MixEnrich, we used another RNA-Seq dataset derived from paired samples of head and neck squamous cell carcinomas (HNSCCs) patients [24].

#### Knowledge-base dataset

In the HNSCCs case study, gene sets were defined using Gene Ontology Biological Process, GO-BP [13, 25]. The GO-BP dataset was retrieved in June 2015 using the org.Hs.eg.db package from Bioconductor [26]. Note, the two terms ‘GO-BP’ and ‘pathway’ are interchangeably used in this present study.

### An Overview of the methodology of N-of-1-*pathways* MixEnrich

**Fig. 1:**
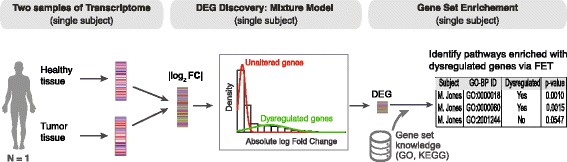
The outline of MixEnrich. Single-subject paired transcriptomes (e.g., healthy and tumor, left panel) are used as the input for the clustering procedure (middle panel). The mixture model clusters all mRNAs of the subject into two groups, determining dysregulated mRNAs. The dysregulated mRNAs are then tested for enrichment into pathways using a Fisher’s Exact Test. FC = fold change; |log_2_FC| = the absolute value of log_2_ transformed fold-change; DEG = differentially expressed mRNAs; FET = Fisher’s Exact Test

We propose a novel method, MixEnrich, under the framework of N-of-1*-pathways*. MixEnrich identifies dysregulated pathways by: (1) clustering mRNAs as unaltered and dysregulated mRNAs and (2) detecting gene sets enriched with dysregulated genes. We named this two-stage procedure MixEnrich for Mixture model clustering followed by an Enrichment analysis. As illustrated in Fig. 1, from a pair of transcriptome derived from a single subject, we constructed a mixture model by modeling the absolute value of log2 transformed fold changes for all mRNAs as a mixture of two distributions: a distribution of dysregulated mRNAs and a distribution of non-dysregulated (unaltered) mRNA expression. We then performed a Fisher’s Exact Test (FET) to determine the over-representation of dysregulated mRNAs (both directions) in each pathway [21].

#### Clustering using the mixture model

For each mRNA, we calculated its absolute value of log_2_ transformed fold change, |log_2_FC|, as |log_2_(E_2_/E_1_)|, where E_1_ is the expression level of this mRNA in condition 1 (e.g., normal tissue) and E_2_ is the expression level in condition 2 (e.g., tumor tissue). Under the mixture model, each mRNA is assumed to belong to a cluster *k* (unaltered mRNA or dysregulated mRNA) with a prior probability *π*_*k*_. The cluster membership of each mRNA is a Bernoulli trial (Eq. 1).

1 $$ {\pi}_k= p\left({Z}_i= k\right),\ {\displaystyle \sum_{k=1}^2}{\pi}_k=1\kern2.25em  i=1,\cdots, G;\  k=1,2 $$

where *Z*_*i*_ is a latent variable and *G* is the total number of mRNAs in the transcriptome. An mRNA for a gene index *i* is a member of cluster *k* when *Z*_i_ is equal to k. We use *x*_i_ to represent |log_2_FC_i_|, and in cluster *k*, the absolute value of log fold change, *x*_*i*_ follows a certain distribution whose parameters need to be estimated. For simplicity, we assumed that the distribution of *x*_*i*_ in each cluster followed a normal distribution, whose probability density function is denoted as φ (Eq. 2).

2 $$ p\left({x}_i\Big|{z}_i= k\right)=\varphi \left({x}_i\Big|{\mu}_k,{\sigma}_k^2\right)\kern1.5em  i=1,\cdots, G; k=1,2 $$

Here *μ*_*k*_ and*σ*_*k*_ are the mean and standard deviation of the normal distribution for the cluster *k*. The marginal distribution of *X* can be obtained by the sum of two weighted normal distributions, hence providing the (discrete) mixture model (Eq. 3).

3 $$ p\left({x}_i\right)={\displaystyle \sum_{k=1}^2}{\pi}_k\varphi \left({x}_i\Big|{\mu}_k,{\sigma}_k^2\right)\kern2.5em  i=1,\cdots, G $$

The estimation of the parameters of the mixture model is implemented by maximum likelihood using an Expectation-Maximization (EM) algorithm [27]. The likelihood that each mRNA belongs to one cluster or the other is assessed by the posterior probability using Bayes rule (Eq. 4).

4 $$ p\left({z}_i= k\Big|{x}_i,{\mu}_1,{\sigma}_1,{\mu}_2,{\sigma}_2\right)=\frac{\pi_k\phi \left({x}_i,\Big|{\mu}_k,{\sigma}_k^2\right)}{{\displaystyle {\sum}_{j=1}^2}{\pi}_j\phi \left({x}_i\Big|{\mu}_j,{\sigma}_j^2\right)\ } $$

We defined an mRNA as dysregulated when its posterior probability of belonging to the dysregulated cluster is above 0.5, where the dysregulated cluster is defined as the cluster with the larger mean.

#### Enrichment of the dysregulated mRNAs

After assigning mRNAs to clusters, a Fisher’s Exact Test (FET) was applied to detect the gene sets (pathways) enriched with dysregulated mRNAs [21]. Assume one pathway consists of *M* mRNAs among which *d* mRNAs are dysregulated; while the entire genome consists of *N* mRNAs among which *D* mRNAs are dysregulated (summarized by a contingency table, Table 2). By this construction, pathway dysregulation is determined relative to the dysregulation of the entire transcriptome as the background. Since different pathways may not be independent due to overlapping mRNAs between them, the *p*-values resulting from FETs were adjusted for multiple hypothesis testing using the approach developed by Benjamini and Yekutieli [28] that accounts for correlated *p*-values.

**Table 2:** Contingency table for Fisher’s Exact Test

	dysregulated mRNAs	unaltered mRNAs	Row sums
mRNAs in target pathway	*d*	*M – d*	*M*
mRNAs not in target pathway	*D - d*	*N - M - D + d*	*N-M*
Column sums	*D*	*N - D*	*N*

### Performance evaluation of the three single-subject methods by simulation

#### Generation of the simulated dataset

Single-subject paired RNA-Seq data were simulated to evaluate the performance of three N-of-1*-pathways* methods: MixEnrich, Wilcoxon, and MD. It has been shown, for biological replicates collected from different subjects, that a negative binomial distribution [29, 30] models the distribution of RNA-Seq read counts more adequately than a Poisson distribution [31, 32], as the negative binomial distribution accounts for the overdispersion (biological variation) of mRNA expression. However, the overdispersion is assumed to be negligible under N-of-1-*pathways* framework since it analyzes the paired samples from the same tissue of the same individual [33]. Therefore, the Poisson distribution is employed to simulate ‘virtual patients’ with various scenarios of dysregulation.

By varying six simulation parameters listed in Table 3, we investigated 107,640 different scenarios of pathway dysregulation. Specifically, for each scenario, we simulated 100 ‘virtual patients’. Each virtual patient has one dysregulated pathway and one unaltered pathway with the same size. The simulation process is as follows:

**Table 3:** Simulation parameters

Parameter	Description of the parameter	Values tested
*bg.FC*	Fold change of dysregulated background mRNAs	{1, 1.3, 1.5, 2}
*bg.dPct*	Percentage of dysregulated mRNAs as noise in the background	{0, 0.01, 0.05, 0.1, 0.2}
*p.S*	Number of mRNAs randomly chosen in the target pathway	{5, 10, [15, 490] by step 25, 500}
*p.dPct*	Percentage of dysregulated mRNAs in the target pathway	{(0, 1] by step 0.05}
*p.FC*	Fold change of mRNAs in the target pathway	{1.3, 1.5, 2}
*p.upPct*	Percentage of up-regulated mRNAs among dysregulated mRNAs in the target pathways	{0, 0.1, 0.2, 0.3, 0.4, 0.5}

1. Estimate the expression mean for every mRNA, *g*, from 55 RNA-Seq normal lung samples downloaded from TCGA (Table 1).
2. Generate a pair of expression values, Y_g1_ and Y_g2_, for each mRNA g in two conditions (normal vs. tumor) using Poisson distribution, Poisson (*λ*_*g*_).
  $$ {Y}_{g1} \sim Poisson\left({\lambda}_g\right) $$
  $$ {Y}_{g2} \sim Poisson\left({\lambda}_g\right) $$
3. Generate a dysregulated pathway:
  - Randomly sample a proportion (*bg.dPct*) of mRNAs, in the second transcriptome (tumor), without replacement, and then replace their values by their corresponding values in the first transcriptome (normal) multiplied by a fold change (*bg.FC*).
  - Designate the target pathway by randomly sampling mRNAs (the number of sampled mRNAs = *p.S*) from the transcriptome without replacement.
  - Randomly sample mRNAs (the number of sampled mRNAs = *p.S* × *p.dPct*) from the target pathway without replacement, and designate the sampled mRNAs as dysregulated.
  - Among the designated dysregulated mRNAs in the target pathway, randomly assign a proportion (*p.upPct*) of these mRNAs as up-regulated. The rest of the designated dysregulated mRNAs are assigned as down-regulated.
  - For the up-regulated mRNAs in the target pathway, replace their values in the second sample (tumor) by their corresponding values in the first sample (normal) multiplied by a fold change (*p.FC*); for the down-regulated mRNAs in the target pathway, replace their values in the second sample (tumor) by their corresponding values in the first sample (normal) divided by a fold change (*p.FC*);
  - Generate an unaltered pathway: randomly sample a proportion (*bg.dPct*) of mRNAs without replacement, and then assign these mRNAs to the non-dysregulated pathway.
  - Repeat Steps 1 – 4 100 times to simulate 100 virtual patients under the given scenario.

#### Comparing the performance of MixEnrich with Wilcoxon and MD

Using the simulated datasets, we compared the proposed method N-of-1*-pathways* MixEnrich with two other single-subject methods: N-of-1-*pathways* Wilcoxon [6] and MD [9]. We evaluated the performance of the three methods by the following measurements:

#### Area under the ROC Curve (AUC)

For each scenario of pathway dysregulation, we calculated an Area Under the receiver operating characteristic Curve (AUC) value as follows: Each scenario corresponds to 100 ‘virtual patients’, and each ‘virtual patient’ possesses one dysregulated pathway and one unaltered pathway. At a given *p*-value threshold, among the 100 dysregulated pathways (*p.dPct* > 0), those identified as dysregulated are true positives (TP) and those identified as unaltered are false negatives (FN). Similarly, among the 100 unaltered pathways (*p.dPct* = 0), those identified as unaltered are true negatives (TN) and those identified as dysregulated are false positives (FP). We calculated the true positive rate (TPR, or sensitivity) and false positive rate (FPR, or Type I error rate) by equation 5 and equation 6:

5 $$ T P R=\frac{TP}{TP+ FN} $$

6 $$ F P R=\frac{FP}{FP+ TN} $$

Receive Operating Characteristic (ROC) curves were generated by plotting FPR against TPR at various *p*-value thresholds. Areas under the ROC curves (AUCs) were computed approximately using Riemann sum in R.

#### Area above the 95% contour curve (AAC_95%_)

We investigated the interaction effect of two simulation parameters, *p.S* and *p.dPct*, on method performance. A contour plot was used to present the joint impact on AUCs induced by the two parameters, *p.S* and *p.dPct*, while fixing the other four simulation parameters listed in Table 3. Each point on the contour plot corresponds to an AUC value of a particular scenario of pathway dysregulation. Then the Area Above the 95% contour Curve (AAC_95%_) was calculated as an overall measure of method accuracy when the two simulation parameters vary simultaneously. Specifically, using color-coded values, we plotted AUCs corresponding to any combination of the two parameters *p.S* and *p.dPct* while fixing the four other parameters, *p.Fc*, *p.upPct*, *bf.FC*, and *bg.dPct*. The horizontal and vertical axes in the contour plot represent the values of *p.S* and *p.dPct,* respectively. AUC values on the contour plot are indicated by color gradient. All points with an AUC value of 95% on the contour plot were connected to construct the 95% curve, demarcating the ACC_95%_ boundary.

### Validation case study of head and neck cell carcinoma patients

We further evaluated the performance of N-of-1-*pathways* MixEnrich, in the context of head and neck squamous cell carcinomas (HNSCCs) (Datasets), using paired RNA-Seq data (tumor vs. healthy) from 45 HNSCC patients. Since a vetted gold standard for HNSCCs does not exist and would require experimentally testing pathways, we established ‘quasi-gold standards’ to evaluate MixEnrich. Forty-five patients were split into two subsets: 30 patients to establish a quasi-gold standard, and 15 testing patients to test the methods. The quasi-gold standard was defined as the dysregulated GO-BP terms identified from the 30 patients using a well-accepted cohort-based method: DESeq (Anders and Huber, 2010) followed by enrichment test (DESeq + Enrichment). DESeq identifies mRNAs differentially expressed between 30 samples of normal tissue and 30 samples of tumor tissue. Nominal *p*-values resulted from DESeq were adjusted for multiplicity via the method proposed by Benjamini and Hochberg to produce FDR_BH_ values. mRNAs with FDR_BH_ < 0.05 were defined as differentially expressed mRNAs (DEGs). Every pathway was then tested for enrichment by a Fisher’s Exact Test [21] to determine the enrichment of DEGs. Since different pathways may share mRNAs and therefore resultant *p*-values are dependent, the multiplicity adjustment developed by Benjamini and Yekutieli was used to calculate FDR_BY_ [28] for adjusting multiple hypothesis testing. The quasi-gold standard was constructed as the set of all pathways with FDR_BY_ < 0.05.

Employing the quasi-gold standard, we compared the accuracy of MixEnrich with that of MD, Wilcoxon, GSEA and DESeq + Enrichment. N-of-1-*pathways* methods, MixEnrich, MD, and Wilcoxon, are single-subject methods and were conducted on every single patient of the 15 testing patients. 15 area under the ROC curves (AUCs) were calculated for each N-of-1-*pathways* methods. Since GSEA and DESeq + Enrichment can only perform on a group of patients, they were evaluated on 50 distinct subsets, which contain 3, 6, or 12 patients, of the 15 testing patients. Taking the subset of 3 patients as an example, 15 testing patients can yield 455 distinct combinations of three patients. To mitigate computational burden, we randomly chose 50 distinct combinations from the 455 combinations as a test set. GSEA and DESeq + Enrichment were conducted on every combination of the 50 distinct patient combinations, which yielded 50 AUCs for each method when compared to the quasi-gold standard. The AUCs resulted from each N-of-1-*pathways* methods and the AUCs resulted from cohort-based methods performed on 3, 6, or 12 patients were plotted by boxplots. With the same strategy, we also evaluated MixEnrich in the context of breast invasive carcinoma (BRCA) (Datasets) using paired RNA-Seq data (tumor vs. healthy) from 112 BRCA patients (Additional file 1: Figure S1).

## Results and Discussion

### Simulation study

To evaluate the performance of N-of-1-*pathways* MixEnrich, we produced synthetic datasets corresponding to 107,640 scenarios of pathway dysregulation by varying six simulation parameters (Table 3). We compared N-of-1-*pathways* MixEnrich with two other single-subject methods, Wilcoxon, and MD, based on (i) the overall performance across all types of dysregulated pathways (Global comparison of the three N-of-1-pathways methods); (2) change in performance as the value of a single simulation parameter varies (MixEnrich is robust against background noise and bidirectional dysregulation), and (3) the change in accuracy as two critical parameters, pathway size (*p.S*) and percentage of the dysregulated mRNAs in the target pathway (*p.dPct*), vary simultaneously (MixEnrich outperforms MD and Wilcoxon when studying the joint effect of pathway size and proportion of dysregulated mRNAs).

#### Global comparison of the three N-of-1-pathways methods

**Fig. 2:**
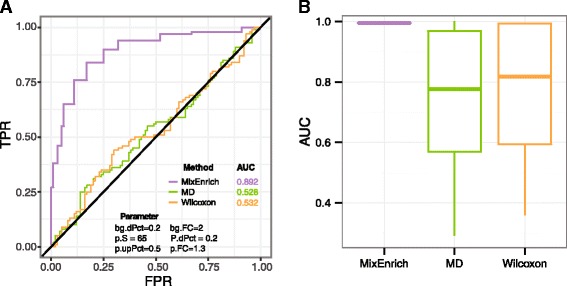
Illustrative ROC curves and comparison of the overall performance of three single-subject methods. MixEnrich is compared to MD and Wilcoxon in overall performance across all simulated pathway dysregulation scenarios via area under ROC curves (AUCs). Panel **a** shows an example of ROC curves for the three methods derived from the following setting: 20% of mRNAs in the background were dysregulated at fold change of 2; 20% of mRNAs in the target pathways (size of 65 genes) were dysregulated at fold change of 1.3 with half of them up-regulated. Each boxplot, in Panel **b**, visualizes all resultant AUCs of the corresponding method across all simulation settings (outliers are not illustrated)

We compared N-of-1-*pathways* MixEnrich with MD and Wilcoxon for their overall performance across all types of pathway dysregulation by combing all 107,640 AUCs (Comparing the performance of MixEnrich with Wilcoxon and MD, Fig. 2). Using Wilcoxon signed-rank test [34], the AUCs of MixEnrich are significantly higher than the ones of N-of-1-*pathways* Wilcoxon (*p*-value < 1 × 10^−10^) and MD (*p*-value < 1 × 10^−10^). This result is further supported by the boxplots (Fig. 2b) comparing the overall performance across all simulated pathway dysregulation scenarios (107,640 AUCs for each method) suggesting that MixEnrich is preferable to Wilcoxon and MD for single-subject transcriptome analysis to evaluate the dynamic change in gene expression in the presence of background noise or to uncover bidirectionally dysregulated pathways, as detailed in the subsequent sections.

#### MixEnrich is robust against background noise and bidirectional dysregulation

**Fig. 3:**
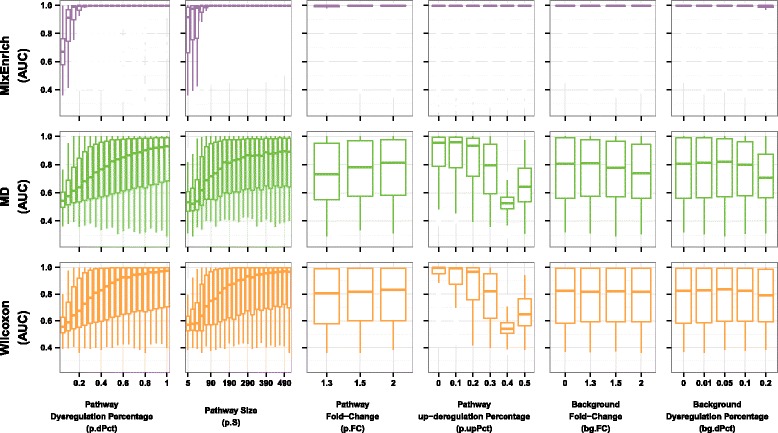
Evaluation of performance as each parameter of the simulation varies Each column corresponds to one simulation parameter (horizontal axis), while each row corresponds to a method (names on the left of the vertical axis). Each panel, defined by the combination of a simulation parameter and a method, contains all 107,640 AUCs resulted from a method. For example, in the panel of pathway dysregulation percentage (*p.dPct*) for N-of-1-*pathways* Wilcoxon, bottom left panel, each boxplot illustrates the distribution of AUCs resulting from Wilcoxon at a fixed value of *p.dPct* (horizontal axis) while varying all the other five simulation parameters. For the sake of clarity, outliers are not shown

We further explored the relative effect of each of the six simulation parameters (Table 3) on the performance of N-of-1-*pathways* MixEnrich in comparaison to Wilcoxon and MD. The boxplots in the fourth column of Fig. 3 confirm that MixEnrich performed uniformly well across all values of *p.upPct* (*p.upPct* = 0, 0.1, 0.2, 0.3, 0.4 or 0.5)*.* On the other hand, the performance of Wilcoxon and MD decreased dramatically as the value of *p.upPct* increased. Unlike MixEnrich, both N-of-1-*pathways* Wilcoxon and MD were designed to identify the pathways only with concordant dysregulation, i.e., dysregulated mRNAs within a pathway are either exclusively up-regulated (*p.upPct* = 1) or exclusively down-regulated (*p.upPct* = 0). Wilcoxon and MD aim to identify the central tendency shift of pathway expression; mRNAs dysregulated in opposing directions counterbalance each other. In contrast, MixEnrich can identify complex bidirectional dysregulation of a pathway since mRNAs dysregulated in both directions contribute additively to the over-representation of a pathway in dysregulated mRNAs.

The increase in percentage of background dysregulated mRNAs (indicative of greater transcriptome noise) does not affect the performance of MixEnrich (Fig. 3, sixth column). Compared to MixEnrich, Wilcoxon and MD are less accurate at various percentages of dysregulated mRNAs in the background (Fig. 3, sixth column). MixEnrich is a competitive model [16] that compares mRNAs in a pathway against the background transcriptome and distinguishes pathways that are significantly more dysregulated than the background transcriptome. In contrast, MD and Wilcoxon are self-contained methods [16], implying that they only use mRNA (gene) expression within a given pathway. Therefore, MixEnrich is expected to have a lower false positive rate when there are a lot of dysregulated mRNAs in the background noise such as biological variation and technical artefacts [16, 35]. However, as the percentage of background noise increases, the performance declines of Wilcoxon and MD are moderate. The data suggest that bidirectional dysregulation decreases the performance of Wilcoxon and MD more severely than the background noise, and therefore the degenerate effect of background noise is hidden by the effect of bidirectional dysregulation (data not shown). Notably, all three methods perform better as the percentage of dysregulated mRNAs in a pathway (*p.dPct*), pathway size (*p.S*), or fold change of the dysregulated mRNAs in a pathway (*p.FC*) increase (Fig. 3, first, second, and third columns).

#### MixEnrich outperforms MD and Wilcoxon when studying the joint effect of pathway size and proportion of dysregulated mRNAs

**Fig. 4:**
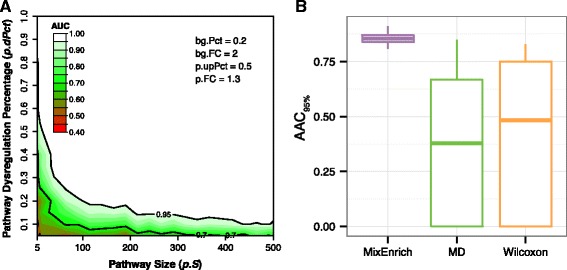
Joint effect of the pathway size and proportion of dysregulated mRNAs on the performance of MixEnrich method. Panel **a** shows an example contour plot of the AUC values under the combination of the four parameters (values are shown at the up-right corner in the contour plot) for MixEnrich. Every point in the contour plot is colored by the AUC value, with the color key shown in the upper left corner of the panel. Points with the same AUC values are connected by contour lines in the plot. We used area above the 95% curve (AAC_95%_; white area above the contour line of AUC = 0.95) as the overall joint performance measure when the two parameters change simultaneously. Panel **b** shows the distribution of every possible AAC_95%_ for each method; each boxplot includes 234 data points; each point in a boxplot corresponds to a specific combination of the four other parameters: *bg.Pct*, *bg.FC*, *p.upPct*, and *p.FC* while allowing *p.S* and *p.dPct* to vary

The number of mRNAs in the pathway (*p.S*) and the proportion of these mRNAs that are dysregulated (*p.dPct*) are two factors most relevant to biology. A comparison (Fig. 4 Panel b) of the AAC_95%_ (Comparing the performance of MixEnrich with Wilcoxon and MD) distributions for the three single-subject methods demonstrates that MixEnrich produced an overall better performance when two parameters, *p.S* and *p.dPct,* chang simultaneously. Using Wilcoxon signed-rank test to compare AAC_95%_, MixEnrich outperformed both N-of-1-*pathways* MD and N-of-1-*pathways* Wilcoxon (*p* <1 × 10^−10^ and *p* <1 × 10^−10^, respectively). MixEnrich obtained an AAC_95%_ > 0.8 for 228 of the 234 tested scenarios while N-of-1-*pathways* MD and N-of-1-*pathways* Wilcoxon yielded AAC_95%_ > 0.8 for 15 and 22 of scenarios, respectively. In the scenarios in which MixEnrich yielded AAC_95%_ < 0.8, the fold change of dysregulated mRNAs was small (1.3 FC) in both the target pathway (*p.FC*) and the background noise (*bg.FC*).

### Validation case study: pathways uncovered by MixEnrich agree with the quasi-gold standard

**Fig. 5:**
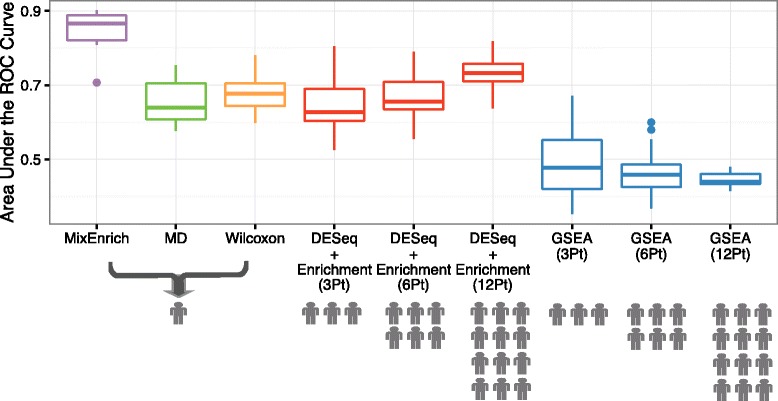
MixEnrich shows higher performance than other single-subject and cohort-based methods (the latter utilized on small samples). Each boxplot corresponding to the N-of-1-*pathways* methods (MixEnrich in purple, MD in green, and Wilcoxon in orange) consists of 15 AUCs resulting from 15 tested patients. Each boxplot corresponding to the cohort-based methods (DESeq + Enrichment in red and GSEA in blue) includes 50 AUCs resulting from 50 distinct subsets of the 15 tested patients (Validation case study of head and neck cell carcinoma patients). Cohort-based methods were performed across 3, 6 and 12 patients (Pt). The number of distinct subjects is shown below the horizontal axis as human icons to further illustrate how many distinct subjects are required in cohort-based analyses to obtain improvements of the AUC (vertical axis). In addition, the three single-subject analyses predict between 200-300 candidate pathways at FDR = 1%, while cohort-based statistics operating on 3 to 12 individuals predict only 50 pathways at FDR = 5% and over 200 at FDR = 20% (data not shown), which explains in part the observed differences in accuracies

We investigated the biological relevance of the dysregulated pathways uncovered by N-of-1-*pathways* MixEnrich using a biological dataset of RNA-seq paired samples (healthy and cancer tissues) derived from head and neck squamous cell carcinoma patients, HNSCCs [24], presented in Table 1. MixEnrich outperforms N-of-1-*pathways* MD and Wilcoxon as well as conventional cohort-based methods GSEA and DESeq + Enrichment in uncovering dysregulated GO-BP terms for HNSCCs. Since it is not feasible to biologically test each pathway to determine the truly dysregulated pathways and unaltered pathways, we conducted DESeq + Enrichment on the 30 patients of HNSCCs cohort to produce a quasi-gold standard (Validation case study of head and neck cell carcinoma patients). DESeq identified 4061 differentially expressed genes from the 30 patients, and a big proportion of these genes were recapitulated by the intermediate step of MixEnrich (Additional file 1: Table S1). The quasi-gold standard consisted of 251 dysregulated GO-BP terms out of the total 3,485 GO-BP terms. MixEnrich achieved higher AUCs (Validation case study of head and neck cell carcinoma patients) in general on predicting the quasi-gold standard in comparison to MD and Wilcoxon (Fig. 5) as well as when compared to AUCs yielded by cohort-based methods conducted across 3, 6 and 12 patients. The superior performance of MixEnrich over cohort-based methods is likely attributed to two reasons: (i) cohort-based methods are underpowered when the sample size is small, and (ii) MixEnrich detects patient-specific signals in addition to the common signals shared among the three patients.

We then tested the hypothesis that single-subject method MixEnrich can capture the individual signals in addition to the common signals shared by all patients. Interestingly, an outlier (patient ID: A6H7) presents in the MixEnrich results, which carries a lower AUC of 0.707. We investigated the dysregulated pathways identified by MixEnrich from patient A6H7 but are not present in the quasi-gold standard (Additional file 1: Table S2). Most of those pathways are related to cell cycle, DNA damage repair, and inflammatory response. Further, all of the GO-BP terms that are identified as dysregulated by MixEnrich from all 15 testing patients exist in the quasi-gold standard (Additional file 1: Table S3).

We also performed another case study using a dataset of matched healthy and cancer RNA-seq samples derived from 112 breast invasive carcinoma patients (Table 1) and again observed the superior performance of MixEnrich (Additional file 1: Figure S1).

### Limitations and future work

As noted in 3.1.3, MixEnrich does not perform well when the FC of dysregulated mRNAs is small in both the background and the target pathway. In addition, the use of a Poisson distribution in the simulation study can be considered a limitation of our work, as negative binomial distribution could have been employed to introduce more noise in the simulated paired samples. Another possible limitation of our study is the choice of the Expectation Maximization (EM) algorithm [27] for estimating the parameters of the mixture model. This algorithm is not guaranteed to converge towards the global optimum. Since MixEnrich operates on the log_2_ transformed mRNA expression fold changes, it may have higher tendency to discover lowly expressed genes as dysregulated, although making inference on gene sets mitigates the bias towards lowly expressed genes (Additional file 1: Table S7). The datasets used in both the simulation study and the validation case studies contain a large amount of lowly expressed genes (Additional file 1: Table S4); the genes annotated to the dysregulated GO-BP terms identified from each of the 15 testing patients have similar distributions compared to the genes annotated all GO-BP terms investigated in the HNSCCs case study (Additional file 1: Table S5-S6). The two-stage process of clustering and enrichment can be viewed as a general framework for paired single-subject analysis. We speculate that more elaborate statistical models could improve the performance of the clustering. Future studies could employ a more general gamma distribution kernel and explore techniques that automatically determine the number of clusters.

Importantly, the simulation study results highlight that MixEnrich detects pathways more dysregulated than the background. This is not addressed by the self-contained methods of MD and Wilcoxon. The fact that self-contained tests do not perform well according to the criteria imposed in the simulation does not invalidate the use of these approaches. Further, self-contained tests are useful to test a small panel of genes such as obtained by real-time PCR.

The current study is based on two paired samples from a single subject. Further improvements and new features of the N-of-1-*pathways* analytic tools can provide more statistical power as more comprehensive N-of-1 experimental studies and assays may be conceived. For example, future studies may include (i) multiple biological and technical replicates of both tumor and control samples from a single subject or (ii) multiple omics measurements beyond the transcriptome (e.g., proteome, methylome, etc.). Future improvements will need to address N-of-1 studies designed with time-series datasets using multi-gene measurement and genomic information based on data derived from normal, treated, and withdrawn treatment samples from a single patient. As single-cell transcriptome datasets from a single patient are increasingly being studied [36], N-of-1-*pathways* framework can be applied and further improved as demonstrated by our recent work in profiling circulating tumor cells using N-of-1-*pathways* MD [10]. As we strive for precision medicine, we must tackle these challenges to accurately provide personal transcriptome analysis at point of care for diagnosis and prognosis.

## Conclusion

Compared to our previously developed N-of-1-*pathways* methods, Wilcoxon and MD, N-of-1-*pathways* MixEnrich is more effective in detecting non-concordant pathway dysregulation, better reflecting what one would expect in biological pathways. Moreover, this novel two-stage competitive gene set testing strategy provides more resistance to background noise, which is ubiquitous in biological systems. Results based on the head and neck squamous cell carcinomas study demonstrate that the dysregulated pathways discovered using MixEnrich overlap highly with the quasi-gold standard compared to the two single-subject methods (Wilcoxon and MD). In addition, we have shown the robust performance of N-of-1-*pathways* MixEnrich operating on single subjects in identifying dysregulated pathways when compared to small-sample, cohort-based methods (DESeq + Enrichment and GSEA).

In this era of precision medicine, it becomes crucial to develop unbiased and personalized transcriptome analytics for single-subject diagnosis and prognosis, rather than using methods that aggregate signals across heterogeneous patients. N-of-1-*pathways* MixEnrich is an innovative framework that bridges this gap by analyzing paired samples, one patient at a time, and is ostensibly extensible to other quantitative ‘omics measurements (e.g., methylome and proteome). MixEnrich is a valuable tool for studying rare and orphan diseases for which sample sizes remain small whereas cohort-based methods are underpowered in that setting. Lastly, the mRNA- and pathway-level analysis performed patient-by-patient by N-of-1-*pathways* MixEnrich offers more interpretable results for biologists and physicians such as dysregulated mRNAs of interest that can be potentially validated and identified as biomarker candidates for diagnosis.

## Additional file

Additional file 1: Figure S1. MixEnrich shows higher performance than other single-subject methods. We repeated the case study using another dataset that contains matched tumor and normal samples for 142 breast invasive carcinoma patients. Each boxplot corresponding to the N-of-1-*pathways* methods (MixEnrich in purple, MD in green, and Wilcoxon in orange) consists of 15 AUCs resulting from 15 testing patients. **Table S1.** Overlap of dysregulated genes (DEG) between the ones in quasi-gold standard and the ones discovered from single patients. **Table S2.** GO-BP terms do not exist in the quasi-gold standard but are identified as dysregulated by MixEnrich from patient A6H7. **Table S3.** GO-BP terms identified as dysregulated by MixEnrich from all 15 head and neck squamous cell carcinoma patients (HNSCCs) patients. **Table S4.** Summary statistics of expression levels for the three data sets (Datasets). According to the first quartile of the three datasets, all three contain a large amount of lowly expressed genes. **Table S5.** Summary statistics of the expression levels for the genes annotated to the dysregulated GO-BPs identified from each of the 15 testing patients. **Table S6.** Summary statistics of the expression levels for the genes annotated to the any GO-BPs investigated in the HNSC validation case study. **Table S7.** True positive rate (TPR) and false positive rate (FPR) of MixEnrich at the final enrichment step (pathways) are improved as compared to the initial clustering step (mRNAs). We randomly chose 1000 pathway dysregulation scenarios from the simulation study (Generation of the simulated dataset). For each dysregulation scenario, we ran MixEnrich and computed TPR and FPR at the enrichment step as described in Comparing the performance of MixEnrich with Wilcoxon and MD. In addition, we computed the TPR and FPR at the clustering step, at which MixEnrich defines a positive case (dysregulated mRNA) as one whose posterior probability of being dysregulated (Eq. 4) is greater than its posterior probability of being unaltered. True positive mRNAs (TP) at the clustering step are the ones that were dysregulated in simulation and also identified as dysregulated by MixEnrich. False positive mRNAs (FP) at this step are the ones that were not dysregulated in simulation but identified as dysregulated by MixEnrich. (DOCX 36 kb)
