# Supplementary material for: N-of-1-pathways MixEnrich: advancing precision medicine via single-subject analysis in discovering dynamic changes of transcriptomes
Source: BMC Med Genomics. 2017 May 24;10(Suppl 1):27. doi: 10.1186/s12920-017-0263-4 (PMC5461551; doi:10.1186/s12920-017-0263-4)
Supplement: Additional file 1: Figure S1. — MixEnrich shows higher performance than other single-subject methods. We repeated the case study using another dataset that contains matched tumor and normal samples for 142 breast invasive carcinoma patients. Each boxplot corresponding to the N-of-1-pathways methods (MixEnrich in purple, MD in green, and Wilcoxon in orange) consists of 15 AUCs resulting from 15 testing patients. Table S1. Overlap of dysregulated genes (DEG) between the ones in quasi-gold standard and the ones discovered from single patients. Table S2. GO-BP terms do not exist in the quasi-gold standard but are identified as dysregulated by MixEnrich from patient A6H7. Table S3. GO-BP terms identified as dysregulated by MixEnrich from all 15 head and neck squamous cell carcinoma patients (HNSCCs) patients. Table S4. Summary statistics of expression levels for the three data sets (Datasets). According to the first quartile of the three datasets, all three contain a large amount of lowly expressed genes. Table S5. Summary statistics of the expression levels for the genes annotated to the dysregulated GO-BPs identified from each of the 15 testing patients. Table S6. Summary statistics of the expression levels for the genes annotated to the any GO-BPs investigated in the HNSC validation case study. Table S7. True positive rate (TPR) and false positive rate (FPR) of MixEnrich at the final enrichment step (pathways) are improved as compared to the initial clustering step (mRNAs). We randomly chose 1000 pathway dysregulation scenarios from the simulation study (Generation of the simulated dataset). For each dysregulation scenario, we ran MixEnrich and computed TPR and FPR at the enrichment step as described in Comparing the performance of MixEnrich with Wilcoxon and MD. In addition, we computed the TPR and FPR at the clustering step, at which MixEnrich defines a positive case (dysregulated mRNA) as one whose posterior probability of being dysregulated (Eq. 4) is greater than its posterior probability of be [file 12920_2017_263_MOESM1_ESM.docx]

| **** |
| --- |
| **Figure S1. MixEnrich shows higher performance than other single-subject methods.** We repeated the case study using another dataset that contains matched tumor and normal samples for 142 breast invasive carcinoma patients. Each boxplot corresponding to the N-of-1-*pathways* methods (MixEnrich in purple, MD in green, and Wilcoxon in orange) consists of 15 AUCs resulting from 15 testing patients. |

**Table S1. Overlap of dysregulated genes (DEG) between the ones in quasi-gold standard and the ones discovered from single patients.**

| **Patient ID** | **# of DEGs identified by MixEnrich** | **# of DEGs identified in quasi-gold standard** | **overlap** | **% of DEGs in quasi-gold standard discovered by MixEnrich** |
| --- | --- | --- | --- | --- |
| 6936 | 3870 | 4061 | 1834 | 45.2% |
| 6938 | 6084 | 4061 | 2311 | 56.9% |
| 6956 | 5145 | 4061 | 2280 | 56.1% |
| 6961 | 4434 | 4061 | 1802 | 44.4% |
| 7101 | 5033 | 4061 | 2212 | 54.5% |
| 7178 | 3871 | 4061 | 1855 | 45.7% |
| 7183 | 3247 | 4061 | 1449 | 35.7% |
| 7235 | 3693 | 4061 | 1869 | 46% |
| 7238 | 4545 | 4061 | 2281 | 56.2% |
| 7250 | 4522 | 4061 | 2048 | 50.4% |
| 7255 | 5138 | 4061 | 2156 | 53.1% |
| 7261 | 4412 | 4061 | 1855 | 45.7% |
| 7432 | 5180 | 4061 | 2191 | 54% |
| A6C4 | 4999 | 4061 | 2104 | 51.8% |
| A6H7 | 3534 | 4061 | 1161 | 28.6% |

**Table S2. GO-BP terms do not exist in the quasi-gold standard but are identified as dysregulated by MixEnrich from patient A6H7.**

| **GO ID** | **description** |
| --- | --- |
| GO:0001701 | in utero embryonic development |
| GO:0043161 | proteasome-mediated ubiquitin-dependent protein catabolic process |
| GO:0072395 | signal transduction involved in cell cycle checkpoint |
| GO:0010498 | proteasomal protein catabolic process |
| GO:0019221 | cytokine-mediated signaling pathway |
| GO:0072413 | signal transduction involved in mitotic cell cycle checkpoint |
| GO:1902402 | signal transduction involved in mitotic DNA damage checkpoint |
| GO:1902403 | signal transduction involved in mitotic DNA integrity checkpoint |
| GO:0071345 | cellular response to cytokine stimulus |
| GO:0001890 | placenta development |
| GO:0071229 | cellular response to acid chemical |
| GO:0006977 | DNA damage response, signal transduction by p53 class mediator resulting in cell cycle arrest |
| GO:2000060 | positive regulation of protein ubiquitination involved in ubiquitin-dependent protein catabolic process |
| GO:0072431 | signal transduction involved in mitotic G1 DNA damage checkpoint |
| GO:1902400 | intracellular signal transduction involved in G1 DNA damage checkpoint |
| GO:0006281 | DNA repair |
| GO:0006997 | nucleus organization |
| GO:1903050 | regulation of proteolysis involved in cellular protein catabolic process |
| GO:1903362 | regulation of cellular protein catabolic process |
| GO:0031570 | DNA integrity checkpoint |
| GO:0044770 | cell cycle phase transition |
| GO:0000075 | cell cycle checkpoint |
| GO:2000058 | regulation of protein ubiquitination involved in ubiquitin-dependent protein catabolic process |
| GO:0002673 | regulation of acute inflammatory response |
| GO:0044772 | mitotic cell cycle phase transition |
| GO:0050731 | positive regulation of peptidyl-tyrosine phosphorylation |
| GO:0007610 | behavior |
| GO:0007093 | mitotic cell cycle checkpoint |
| GO:0051437 | positive regulation of ubiquitin-protein ligase activity involved in regulation of mitotic cell cycle transition |
| GO:0006521 | regulation of cellular amino acid metabolic process |
| GO:0006998 | nuclear envelope organization |
| GO:0009792 | embryo development ending in birth or egg hatching |
| GO:0043009 | chordate embryonic development |
| GO:0002526 | acute inflammatory response |
| GO:0031145 | anaphase-promoting complex-dependent proteasomal ubiquitin-dependent protein catabolic process |
| GO:0001503 | ossification |
| GO:0007346 | regulation of mitotic cell cycle |
| GO:0048520 | positive regulation of behavior |
| GO:0000077 | DNA damage checkpoint |
| GO:0044773 | mitotic DNA damage checkpoint |
| GO:0051351 | positive regulation of ligase activity |
| GO:0042176 | regulation of protein catabolic process |
| GO:0006956 | complement activation |
| GO:0002521 | leukocyte differentiation |
| GO:0050730 | regulation of peptidyl-tyrosine phosphorylation |
| GO:0048534 | hematopoietic or lymphoid organ development |
| GO:0040017 | positive regulation of locomotion |
| GO:0033993 | response to lipid |
| GO:0045596 | negative regulation of cell differentiation |
| GO:0051439 | regulation of ubiquitin-protein ligase activity involved in mitotic cell cycle |
| GO:0042517 | positive regulation of tyrosine phosphorylation of Stat3 protein |
| GO:0070098 | chemokine-mediated signaling pathway |
| GO:0007600 | sensory perception |
| GO:0001101 | response to acid chemical |
| GO:0046427 | positive regulation of JAK-STAT cascade |
| GO:0052547 | regulation of peptidase activity |
| GO:0002683 | negative regulation of immune system process |
| GO:0030278 | regulation of ossification |
| GO:0051443 | positive regulation of ubiquitin-protein transferase activity |
| GO:2000147 | positive regulation of cell motility |
| GO:0031571 | mitotic G1 DNA damage checkpoint |
| GO:0044783 | G1 DNA damage checkpoint |
| GO:0044819 | mitotic G1/S transition checkpoint |

**Table S3. GO-BP terms identified as dysregulated by MixEnrich from all 15 head and neck squamous cell carcinoma patients (HNSCCs) patients.**

| **GO ID** | **Description** | **In quasi-gold standard** |
| --- | --- | --- |
| GO:0000375 | RNA splicing, via transesterification reactions | Yes |
| GO:0000377 | RNA splicing, via transesterification reactions with bulged adenosine as nucleophile | Yes |
| GO:0000398 | mRNA splicing, via spliceosome | Yes |
| GO:0001501 | skeletal system development | Yes |
| GO:0006396 | RNA processing | Yes |
| GO:0006397 | mRNA processing | Yes |
| GO:0006511 | ubiquitin-dependent protein catabolic process | Yes |
| GO:0007606 | sensory perception of chemical stimulus | Yes |
| GO:0007608 | sensory perception of smell | Yes |
| GO:0008380 | RNA splicing | Yes |
| GO:0009593 | detection of chemical stimulus | Yes |
| GO:0009790 | embryo development | Yes |
| GO:0016071 | mRNA metabolic process | Yes |
| GO:0019941 | modification-dependent protein catabolic process | Yes |
| GO:0022613 | ribonucleoprotein complex biogenesis | Yes |
| GO:0030198 | extracellular matrix organization | Yes |
| GO:0032963 | collagen metabolic process | Yes |
| GO:0034660 | ncRNA metabolic process | Yes |
| GO:0043062 | extracellular structure organization | Yes |
| GO:0043632 | modification-dependent macromolecule catabolic process | Yes |
| GO:0044236 | multicellular organismal metabolic process | Yes |
| GO:0044259 | multicellular organismal macromolecule metabolic process | Yes |
| GO:0050906 | detection of stimulus involved in sensory perception | Yes |
| GO:0050907 | detection of chemical stimulus involved in sensory perception | Yes |
| GO:0050911 | detection of chemical stimulus involved in sensory perception of smell | Yes |
| GO:0051603 | proteolysis involved in cellular protein catabolic process | Yes |
| GO:0072358 | cardiovascular system development | Yes |
| GO:0072359 | circulatory system development | Yes |

**Table S4 Summary statistics of expression levels for the three data sets (Datasets). According to the first quartile of the three datasets, all three contain a large amount of lowly expressed genes.**

| **Dataset** | **Min** | **1^st^ Quartile** | **Median** | **Mean** | **3^rd^ Quartile** | **Max** |
| --- | --- | --- | --- | --- | --- | --- |
| Dataset I | 0 | 18.65 | 393.9 | 1730 | 1454 | 902500 |
| Dataset III | 0 | 36.74 | 563.2 | 2715 | 2112 | 1035000 |
| Dataset II | 0 | 0.8576 | 13.89 | 48.78 | 48.38 | 5651 |

**Table S5. Summary statistics of the expression levels for the genes annotated to the dysregulated GO-BPs identified from each of the 15 testing patients.**

| **Patient ID** | **Min** | **1^st^ Quartile** | **Median** | **Mean** | **3^rd^ Quartile** | **Max** |
| --- | --- | --- | --- | --- | --- | --- |
| 6936 | 0 | 172.4 | 1260 | 4633 | 3607 | 908500 |
| 6938 | 0 | 175.7 | 1186 | 4207 | 3343 | 908500 |
| 6956 | 0 | 203 | 1264 | 4480 | 3523 | 908500 |
| 6961 | 0 | 156.7 | 1177 | 4370 | 3418 | 908500 |
| 7101 | 0 | 193.8 | 1206 | 4296 | 3407 | 908500 |
| 7178 | 0 | 227.2 | 1357 | 4691 | 3754 | 908500 |
| 7183 | 0 | 203.4 | 1223 | 4564 | 3487 | 908500 |
| 7235 | 0 | 171.8 | 1259 | 4631 | 3589 | 908500 |
| 7238 | 0 | 170.4 | 1255 | 4609 | 3613 | 908500 |
| 7250 | 0 | 206.1 | 1236 | 4144 | 3461 | 908500 |
| 7255 | 0 | 186.8 | 1216 | 4362 | 3467 | 908500 |
| 7261 | 0 | 219.8 | 1308 | 4608 | 3645 | 908500 |
| 7432 | 0 | 169.6 | 1223 | 4481 | 3508 | 908500 |
| A6C4 | 0 | 189 | 1236 | 4384 | 3471 | 908500 |
| A6H7 | 0 | 193.8 | 1274 | 5219 | 3881 | 781000 |

**Table S6. Summary statistics of the expression levels for the genes annotated to the any GO-BPs investigated in the HNSC validation case study**

| **Min.** | **1^st^ Quartile** | **Median** | **Mean** | **3^rd^ Quartile** | **Max** |
| --- | --- | --- | --- | --- | --- |
| 0 | 180.6 | 1146 | 4004 | 3196 | 908500 |

**Table S7. True positive rate (TPR) and false positive rate (FPR) of MixEnrich at the final enrichment step (pathways) are improved as compared to the initial clustering step (mRNAs).** We randomly chose 1000 pathway dysregulation scenarios from the simulation study (**Generation of the simulated dataset**). For each dysregulation scenario, we ran MixEnrich and computed TPR and FPR at the enrichment step as described in **Methods Comparing the performance of MixEnrich with Wilcoxon and MD**. In addition, we computed the TPR and FPR at the clustering step, at which MixEnrich defines a positive case (dysregulated mRNA) as one whose posterior probability of being dysregulated (**Eq. 4**) is greater than its posterior probability of being unaltered. True positive mRNAs (TP) at the clustering step are the ones that were dysregulated in simulation and also identified as dysregulated by MixEnrich. False positive mRNAs (FP) at this step are the ones that were not dysregulated in simulation but identified as dysregulated by MixEnrich.

|  | **TPR** (standard deviation) | **FPR** (standard deviation) |
| --- | --- | --- |
| **Clustering step** | 0.643 (0.364) | 0.227 (0.037) |
| **Enrichment step** | 0.906(0.24) | 0.03 (0.019) |
